# Comparison of Laparoscopy and Laparotomy for Para-Aortic Lymphadenectomy in Women With Presumed Stage I–II High-Risk Endometrial Cancer

**DOI:** 10.3389/fonc.2020.00451

**Published:** 2020-04-07

**Authors:** E Sun Paik, Seung Hun Baek, Jun Hyeok Kang, Soo Young Jeong, Myeong Seon Kim, Woo Young Kim, Yoo-Young Lee, Chel Hun Choi, Jeong-Won Lee, Byoung-Gie Kim, Duk-Soo Bae, Tae-Joong Kim

**Affiliations:** ^1^Department of Obstetrics and Gynecology, Kangbuk Samsung Hospital, Sungkyunkwan University School of Medicine, Seoul, South Korea; ^2^Department of Obstetrics and Gynecology, Samsung Medical Center, Sungkyunkwan University School of Medicine, Seoul, South Korea; ^3^Department of Obstetrics and Gynecology, Saint Vincent's Hospital, The Catholic University of Korea, Suwon, South Korea

**Keywords:** endometrial cancer, laparoscopy, lymphadenectomy, para-aortic lymph node, postoperative complications

## Abstract

**Objective:** To compare laparoscopic surgery to laparotomy for harvesting para-aortic lymph nodes in presumed stage I–II, high-risk endometrial cancer patients.

**Methods:** Patients with histologically proven endometrial cancer, presumed stage I-II with high-risk tumor features who had undergone hysterectomy, bilateral salpingoophorectomy, or pelvic and para-aortic lymphadenectomy by either laparoscopy or laparotomy in Samsung Medical Center from 2005 to 2017 were retrospectively investigated. The primary outcome was para-aortic lymph node count. Secondary outcomes were pelvic lymph node count, perioperative events, and postoperative complications.

**Results:** A total of 90 patients was included (35 for laparotomy, 55 for laparoscopy) for analysis. The mean (±SD) para-aortic lymph node count was 10.66 (±7.596) for laparotomy and 10.35 (±5.848) for laparoscopy (*p* = 0.827). Mean pelvic node count was 16.8 (±6.310) in the laparotomy group and 16.13 (±7.626) in the laparoscopy group (*p* = 0.664). Lower estimated blood loss was shown in the laparoscopy group. There was no difference in perioperative outcome between the groups. Additional multivariate analysis showed that survival outcome was not affected by surgical methods in presumed stage I-II, high-risk endometrial cancer patients.

**Conclusions:** Study results demonstrate comparable para-aortic lymph node count with less blood loss in laparoscopy over laparotomy. In women with presumed stage I-II, high-risk endometrial cancer, laparoscopy is a valid treatment modality.

## Introduction

Endometrial carcinoma is the most rapidly increasing female genital tract malignancy. In 2018, it was the sixth most common malignancy in females, accounting for 382,069 new cases world-wide (1). In Korea, the incidence of newly diagnosed endometrial cancer consistently increased in all age groups from 1999 to 2015 (2). Endometrial cancer usually occurs in women after menopause, and most cases present with early-stage disease due to frequent symptoms of abnormal vaginal bleeding, which leads to early detection.

The European Society for Medical Oncology (ESMO), European Society of Gynaecological Oncology (ESGO), and European Society for Radiotherapy and Oncology (ESTRO) guidelines recommend that treatments of high-intermediate risk, high risk, and/or advanced endometrial cancer should include surgical treatment with hysterectomy, bilateral salpingo-oophorectomy with additional pelvic lymph node dissection (PLND), and para-aortic lymph node dissection (PALND) (3, 4). The survival effect of para-aortic lymphadenectomy in endometrial cancer (SEPAL) study demonstrated a significant benefit of pelvic and para-aortic lymphadenectomy on survival in endometrial cancer patients with intermediate or high risk of recurrence (5). The results of lymphadenectomy provide physicians relevant information for adjuvant treatment in the clinical field.

Surgery for endometrial cancer was traditionally performed with laparotomy, but minimally invasive surgery has increased gradually due to lower perioperative and postoperative complication rates and shorter hospital stays without differences in survival outcomes (6, 7). Previously, large-scale randomized trials were conducted for comparison between laparoscopic staging operation for endometrial cancer and laparotomy, but there were no detailed results regarding utility of laparoscopic procedure for systematic lymph node dissection, specifically for the PALND which can be more complex procedure (8, 9).

In this study, we aimed to compare laparoscopy with laparotomy for PALND in patients with presumed stage I-II, high-risk endometrial cancer. The primary outcome of this study was number of harvested para-aortic lymph nodes, and secondary outcomes for study were pelvic node count and perioperative/postoperative surgical outcomes. Additionally, we compared overall survival and recurrence for two patient groups.

## Materials and Methods

### Patients

After obtaining the Institutional Review Board (IRB) approval (No. 2019-07-133-002), we retrospectively reviewed data from all patients with primary endometrial cancer who were surgically treated (laparoscopy and laparotomy) at Samsung Medical Center from January 2005 to December 2017. We investigated data from electronic medical records and included patients based on the following criteria: (1) histologically confirmation of endometrial cancer, (2) presumed stage I or II [International Federation of Gynecology and Obstetrics (FIGO)], (3) pre-operatively evaluated as high-risk tumor [one of following features; non-endometrioid type/endometrioid type FIGO grade 3/endometrioid type FIGO grade 2 with >50% myometrial invasion or invasion in the cervical stroma, evaluated by trans-vaginal ultrasound and/or magnetic resonance imaging (MRI) of pelvis] (10). Criteria were based on concept of using clinically predicted stage before surgery as in actual practice by preoperative evaluation, and pathologic findings after surgical staging (lymphovascular space invasion, lymph node metastasis), were not included in inclusion criteria. Exclusion criteria were ongoing anti-tumor treatment, pre-operative imaging indicating extrauterine spread, and disseminated disease diagnosed during surgery. After diagnostic endometrial biopsy by curettage, all patients had undergone pre-operative evaluation, blood tests, and imaging studies (computed tomography (CT) of the chest and abdomen/pelvis and trans-vaginal ultrasound and/or MRI of the pelvis).

### Surgical Management

Surgical staging for endometrial cancer was performed by laparoscopy (four-port conventional) or laparotomy (midline incision). In our institution, a staging operation was routinely performed according to the Korean Gynecologic Oncology Group (KGOG) surgical manual (11). Regarding lymphadenectomy, pelvic lymph nodes removal was performed from the distal one-half of the common iliac artery to the circumflex iliac vein, and nodal tissue was removed at anterior of the obturator nerve and around the iliac vessels. The para-aortic lymph nodes indicated those covering the vena cava (right para-aortic), middle part between the vena cava and aorta, and to the left of the aorta (left para-aortic). The cephalad border of the para-aortic lymph node was commonly, but not limited to, the inferior mesenteric artery (IMA). The distal border was the midpoint of the common iliac artery (8). Laparoscopic PALND was performed after elevating the IMA for identification of the left ureter. PALND proceeded to the left of the aorta and down the lateral aspect of the left common iliac artery to the midpoint. Six surgeons who specialize in gynecologic oncology in our institution performed surgical staging. All six surgeons were all skilled for laparoscopy and laparotomy procedures in gynecologic surgery, and they were all trained as gynecologic oncologists in major institution in Korea. The lymph node specimens were sent to the pathology department for histological evaluation which was performed by gynecologic expert pathologists.

### Outcomes

The primary outcome measure was the number of harvested para-aortic lymph nodes. Secondary outcomes were number of pelvic nodes and perioperative/postoperative outcomes. Operation time was designated as the time (minutes) from first incision of the operation to last closure of the skin. Postoperative complications were investigated until 30 days after surgery, and readmissions within 30 days from surgery were investigated. Length of hospital stay was measured (in days) for initial operation, and in the case of readmission, hospital days after readmission were not included. Additionally, survival outcomes, recurrence-free survival (RFS) and overall survival (OS), were investigated for two surgical methods. RFS was described as the time (months) from surgery to the recurrence or last follow-up. OS was defined as the time (months) from surgery to date of death or last follow-up.

### Statistical Analysis

For purposes of this study, summary statistics were used for description of the data. Median (range) or mean (standard deviation) were used to describe continuous variables. Categorical variables were shown as frequency (percentage). After confirmation of normal distributions with the Shapiro–Wilk test, the Mann–Whitney test was performed to compare median values, and Student's *t*-test was performed to compare mean values. Fisher's exact test or χ^2^ test was performed for analysis of the distribution of characteristics. The Cox proportional hazards model was used for univariate and multivariate analyses to evaluate the prognostic significance of clinicopathologic features for RFS and OS. For multivariate analysis, a stepwise backward method was used. Variables associated with RFS and OS with a significance level of *P* < 0.05 in univariate analysis were selected as possible variables for multivariate logistic regression analysis. A 95% confidence interval (CI) was used to quantify the correlation between RFS or OS and each independent feature. All *P*-values were two-sided, and *P* < 0.05 were regarded as statistically significant. All statistical analyses were accomplished using IBM SPSS Statistics software Version 21.0 (IBM, Armonk, New York, USA).

## Results

### Patients Characteristics

This study analyzed 90 presumed stage I–II, high-risk endometrial cancer patients treated in Samsung Medical Center from 2005 to 2017. Within this patient group, 35 patients underwent laparotomy, and 55 patients had laparoscopic surgical staging. Basal characteristics are shown in Table 1. There were significant differences in age among patients undergoing the two surgical methods, but there were no statistically significant differences for other variables between the two groups.

**Table 1:** Baseline demographics and clinicopathologic characteristics of endometrial cancer patients by surgical technique (n = 90).

**Characteristic**	**Laparotomy (*n* = 35)**	**Laparoscopy (*n* = 55)**	***p*-value**
**Age, years**			
Median (range)	59 (39–77)	53 (35–70)	0.002
**BMI, kg/m**^**2**^			
Median (range)	23.4 (18.3–38.0)	23.8 (17.4–39.3)	0.699
**Menopause**			0.072
Yes	27 (77.1)	33 (60.0)	
No	8 (22.9)	22 (40.0)	
**Stage (presumed by preoperative evaluation)**			0.322
IA	16 (45.7)	33 (60.0)	
IB	13 (37.1)	18 (32.7)	
II	6 (17.2)	4 (7.3)	
**Histology**			0.558
Endometrioid	29 (82.9)	41 (74.5)	
Papillary serous	1 (2.9)	2 (3.6)	
Clear cell	3 (8.6)	2 (3.6)	
Others	2 (5.8)	10 (18.2)	
**Grade**			0.261
2	4 (11.4)	3 (5.5)	
3	31 (88.6)	52 (94.5)	
**Pre-operative serum CA-125 level, U/mL**			0.135
Median (range)	11.8 (2.0–408)	10.4 (3.0–145)	
**Myometrial invasion**			0.084
No	7 (20.0)	18 (32.7)	
Superficial	3 (8.6)	0	
Inner half	11 (31.4)	20 (36.4)	
Outer half	11 (31.4)	16 (29.1)	
Full	3 (8.6)	1 (1.8)	
**Lymphovascular space invasion**			0.298
No	20 (57.1)	38 (69.1)	
Yes	15 (42.9)	17 (30.9)	
**Adjuvant treatment**			>0.999
Chemotherapy	2 (5.7)	3 (5.5)	
Radiotherapy	21 (60.0)	34 (61.8)	
Concurrent chemo-radiotherapy	1 (2.9)	2 (3.6)	
No adjuvant therapy	11 (31.4)	16 (29.1)	

*BMI, body mass index; CA-125, cancer antigen 125*.

### Analysis of PALND and PLND

Regarding number of harvested para-aortic lymph nodes [mean (±SD)], no difference between laparoscopy [10.35 (±5.848)], and laparotomy [10.66 (±7.596)] (*p* = 0.827) was shown (Table 2). Also, pelvic lymph node count was not different between the two groups [laparoscopy16.13 (±7.626) vs. laparotomy 16.8 (±6.310), *p* = 0.664]. Lymph node metastasis was shown only in laparotomy group (pelvic 14.3%, para-aortic 8.65%). In this study, patients were included according to clinical FIGO stage I–II, which was presumed by preoperative evaluation, and patients with advanced stage disease due to lymph node metastasis in pathologic staging were inevitably included.

**Table 2:** Number of lymph nodes harvested through systemic lymphadenectomy and prevalence of lymph node metastasis by surgical modality.

**Lymph node region**	**Laparotomy (*n* = 35)**	**Laparoscopy (*n* = 55)**	***p*-value**
**Para-aortic region**			
Median (range)	9 (2–31)	10 (2–23)	0.734
Mean (±SD)	10.66 (7.596)	10.35 (5.848)	0.827
**Pelvic region**			
Median (range)	16 (5–32)	15 (3–41)	0.350
Mean (±SD)	16.8 (6.310)	16.13 (7.626)	0.664
**Lymph node metastasis**, ***n*** **(%)**			
Pelvic	5 (14.3)	0	0.007
Para-aortic	3 (8.6)	0	0.056

*SD, standard deviation*.

### Perioperative and Postoperative Outcomes

Comparison of perioperative and postoperative outcomes between two groups are shown in Table 3. There was significantly less estimated total blood loss in the laparoscopy group than in the laparotomy group (200 vs. 350 mL, *p* = 0.001). Notably, there was need for blood transfusion in 14.3% of the laparotomy cases compared to none in the laparoscopy group. No conversion to laparotomy was shown in the laparoscopy group. Regarding operation time and length of hospital stay, no differences between two groups were shown. There were no differences in intraoperative complication events (5.5 vs. 2.9%, *p* = 0.456) or postoperative 30 day complications (27.3 vs. 34.3%, *p* = 0.196) between the two groups. No patient in either group experienced readmission within 30 days, and no mortality was shown within 30 days after surgery.

**Table 3:** Perioperative and postoperative outcomes per surgical modality.

**Outcomes**	**Laparotomy (*n* = 35)**	**Laparoscopy (*n* = 55)**	***p*-value**
**Operation time, minutes**			0.095
Median (range)	245 (180–502)	267 (144–727)	
**Estimated total blood loss, mL**			0.001
Median (range)	350 (50–800)	200 (50–500)	
**Blood transfusion, no. (%)**			0.007
No	30 (85.7)	55 (100)	
1 pint	2 (5.7)	0	
2 pint	2 (5.7)	0	
4 pint	1 (2.9)	0	
**Length of hospital stay, days**			0.104
Median (range)	10 (6–86)	9 (6–20)	
**Intraoperative adverse events, n (%)**	1 (2.9)	3 (5.5)	0.456
Ureter injury	1 (2.9)	0	
Bladder injury	0	2 (3.6)	
IVC injury	0	1 (1.8)	
**Postoperative complications within 30 days**, ***n*** **(%)**	12 (34.3)	15 (27.3)	0.196
Fever	4 (11.4)	3 (5.5)	
Ileus	4 (11.4)	10 (18.2)	
Surgical wound infection	4(11.4)	1 (5.6)	
Leg pain	0	1 (1.8)	

*IVC, inferior vena cava*.

### Survival Outcomes

In addition to comparison of number of lymph nodes and perioperative complications, survival comparison was performed. After a median follow-up of 61.6 months (range 3.0–136.9 months), there were 17 patients with recurrences, and 10 deaths (Table 4). Regarding recurrence events, difference between the two groups was not significant (*p* = 0.269), but there was a significant difference regarding death events (*p* = 0.043). Kaplan–Meier plots for RFS and OS are shown in Supplementary Material 1. Unevenly distributed ages between groups and discrepancy between preoperative clinical stage and result of pathologic evaluation (lymph node metastasis) may be reasons for the survival difference. In multivariate analysis for OS, type of surgical procedure was not a significant factor for OS [HR (95% CI) 0.444 (0.097–2.030), *p* = 0.295, Table 5]. Results of multivariate analysis demonstrated that survival outcome was not affected by surgical method in presumed stage I–II, high-risk endometrial cancer patients.

**Table 4:** Survival comparison by patients group with laparoscopy and laparotomy.

**Characteristic**	**Laparotomy (*n* = 35)**	**Laparoscopy (*n* = 55)**	***p*-value**
**Recurrence event**	9 (25.7)	8 (14.5)	0.269
**Recurrence-free survival, months**			
Median (range)	51.5 (3.0–136.9)	60.5 (3.0–135.4)	0.646
**Death event**	7 (20.0)	3 (5.5)	0.043
**Overall-survival, months**			
Median (range)	56.0 (3.0–136.9)	61.6 (3.0–136.9)	0.734

**Table 5:** Univariate and multivariate Cox proportional hazards analysis for recurrence-free survival (RFS), and overall survival (OS) to adjust risk associated with prognostic features.

**Variables**	**Univariate**		**Multivariate**	
	**HR (95% CI)**	***p*-value**	**HR (95% CI)**	***p*-value**
**RFS**				
Age	1.009 (0.953–1.068)	0.769	N/A	
Clinical stage (I vs. II)	3.587 (1.160–11.090)	0.027	2.303 (0.565–9.381)	0.244
Histology (Endometrioid vs. non-endometrioid)	0.791 (0.227–2.753)	0.712	N/A	
Grade (2 vs. 3)	0.634 (0.145–2.773)	0.545	N/A	
Myometrial invasion (<1/2 vs. >1/2)	4.167 (1.537–11.300)	0.005	3.679 (1.337–10.122)	0.012
Lymphovascular space invasion (no vs. yes)	3.898 (1.440–10.552)	0.007	1.988 (0.659–5.997)	0.222
Cervical stromal invasion (no vs. yes)	3.184 (1.171–8.662)	0.023	0.534 (0.118–2.412)	0.415
LN metastasis (no vs. yes)	8.341 (2.656–26.201)	<0.001	6.677 (2.073–21.509)	0.001
Type of surgical procedure (Laparotomy vs. Laparoscopy)	0.532 (0.205–1.380)	0.195	N/A	
**OS**				
Age	1.076 (0.998–1.077)	0.058	N/A	
Clinical stage (I vs. II)	5.528 (1.412–21.513)	0.014	1.791 (0.325–9.853)	0.503
Histology (Endometrioid vs. non-endometrioid)	2.648 (0.747–9.392)	0.132	N/A	
Grade (2 vs. 3)	0.719 (0.091–5.677)	0.719	N/A	
Myometrial invasion (<1/2 vs. >1/2)	2.341 (0.677–8.103)	0.179	N/A	
Lymphovascular space invasion (no vs. yes)	3.244 (0.914–11.520)	0.069	N/A	
Cervical stromal invasion (no vs. yes)	6.053 (1.744–21.004)	0.005	3.794 (0.903–15.949)	0.069
LN metastasis (no vs. yes)	9.684 (2.474–37.907)	0.001	4.587 (0.946–22.244)	0.059
Type of surgical procedure (Laparotomy vs. laparoscopy)	0.250 (0.064–0.966)	0.044	0.444 (0.097–2.030)	0.295

*n = 90. RFS, recurrence-free survival; OS, overall-survival; HR, hazard ratio; CI, confidential interval; LN, lymph node; N/A, not available*.

## Discussion

In this study, we concluded that laparoscopy is an acceptable surgical method for harvesting para-aortic lymph nodes in presumed stage I–II high-risk endometrial cancer. Between laparoscopy and laparotomy groups, number of harvested para-aortic lymph nodes was comparable, as well as number of pelvic lymph nodes. Less blood loss was shown in the laparoscopy group, and there were no significant differences in operation time, length of hospital stay, and intraoperative and postoperative complications between the two groups. For survival comparison, no meaningful results were drawn, possibly due to unevenly distributed characteristics and discrepancies between preoperative clinical stage and result of pathologic evaluation (lymph node metastasis). Multivariate analysis revealed that survival outcome was not affected by surgical method in presumed stage I–II, high-risk endometrial cancer patients.

The effect of PALND in endometrial cancer was shown in a previous retrospective cohort study (SEPAL study) (5). In SEPAL study, result of multivariate analysis had shown that PLND and PALND reduced the risk of death compared with that in the PLND only group. Thus, PALND and PLND are recommended for patients with endometrial cancer with intermediate or high risk of recurrence. In a large-scale, randomized trial comparing laparoscopy and laparotomy in endometrial cancer (Gynecologic Oncology Group Study LAP2 trial) (8), results showed that laparoscopic surgical staging for endometrial cancer is feasible and safe in regards to postoperative outcomes which resulted in fewer complications and shorter hospital stays. The LAP2 trial included all stages of endometrial cancer compared to high-risk, presumed stage I–II in our study. In LAP2 trial, pelvic and para-aortic lymph nodes were not removed in 8% of patients of laparoscopy and 4% of laparotomy. Lymph node metastases were found in 9% of patients and were similar in both groups. In present study, lymph node metastasis was present in only the laparotomy group, possibly due to uneven randomization between groups. This may have resulted in survival differences in the current study. Previously, number of studies had shown feasibility of minimally invasive surgery in lymphadenectomy of gynecologic cancers (12–14). A recent randomized trial in Sweden that compared robotic-assisted laparoscopy and laparotomy for infrarenal PALND in early stage high-risk endometrial cancer showed non-inferiority in para-aortic lymph node count, similar complication rates, shorter hospital length, and lower total cost for robotic-assisted laparoscopy over laparotomy (15). Inclusion criteria and primary outcomes were similar to those of the current study, but our study compared a broader field of laparoscopy. We did not compare health care costs, and the results of the previous study may not be universal because medical expenses may vary depending on insurance system of each country.

The strength of the current study is that analysis was performed for data of the procedure (PALND) in a specific group of patients (stage I–II, high-risk endometrial cancer patients) that had not been thoroughly investigated so far. Using specific patient groups for comparison, outcomes of procedures could be more clearly determined. However, a number of potential limitations exist in the current study. The study had used data that were retrospectively collected for analysis. Therefore, it may be biased by patients not-randomly assigned, and incomplete data collection. Inclusion criteria were based on clinically presumed stage as in actual practice by preoperative evaluation and pathologic findings after surgical staging (lymphovascular space invasion, lymph node metastasis), which could be risk factor for adjuvant treatment, were not included. This could be potential bias of patients' selection. Also, this may have led to unevenly distributed characteristics between two groups, and this made it difficult to obtain a meaningful result in comparing the survival of the two groups. Kaplan–Meier plot for OS showed a significant difference between two surgical groups, and we assumed that discrepancy between preoperative clinical stage and result of pathologic evaluation (lymph node metastasis) may be reasons for the survival difference. However, since the primary outcome of this study was a comparison of surgical outcomes, the difference in characteristics between the two groups may not be a problem, and multivariate analysis results demonstrated that OS was not affected by surgical method in this patients group. In addition to limitations and biases associated with its retrospective nature, this study resulted from data of a small number of patients for comparisons. A further large-scale study will provide more meaningful results.

Despite these limitations, we demonstrated that laparoscopy is an acceptable surgical method for harvesting para-aortic lymph nodes in presumed stage I–II, high-risk endometrial cancer patients. Study results showed a similar number of harvested lymph nodes, no significant differences in intraoperative and postoperative complications between laparoscopy and laparotomy, and less blood loss in the laparoscopy group.

## Data Availability Statement

The datasets for this article are not publicly available because IRB of Samsung Medical Center restricted providing raw data of this data to public for privacy issues. Requests to access the datasets should be directed to Review committee of Research Data under IRB in Samsung Medical Center.

## Ethics Statement

This study was approved by the institutional review boards of Samsung Medical Center in accordance with the Declaration of Helsinki and the International Conference on Harmonization Good Clinical Practice guidelines.

## Author Contributions

EP, SB, and T-JK contributed to the conception and design of the study. SB, JK, SJ, and MK organized the database. EP and T-JK performed the statistical analysis. EP and SB wrote the first draft of the manuscript. WK, Y-YL, CC, J-WL, BGK, and D-SB wrote sections of the manuscript. All authors contributed to manuscript revision, read, and approved the submitted version.

### Conflict of Interest

The authors declare that the research was conducted in the absence of any commercial or financial relationships that could be construed as a potential conflict of interest.

## Supplementary Material

Supplementary Material 1 Kaplan–Meier curves for **(A)** recurrence-free survival and **(B)** overall survival by laparoscopy and laparotomy in presumed early-stage, high-risk endometrial cancer patients.
