# Supplementary figures and images for: Comparison of Laparoscopy and Laparotomy for Para-Aortic Lymphadenectomy in Women With Presumed Stage I–II High-Risk Endometrial Cancer
Source: Front Oncol. 2020 Apr 7;10:451. doi: 10.3389/fonc.2020.00451 (PMC7155754; doi:10.3389/fonc.2020.00451)

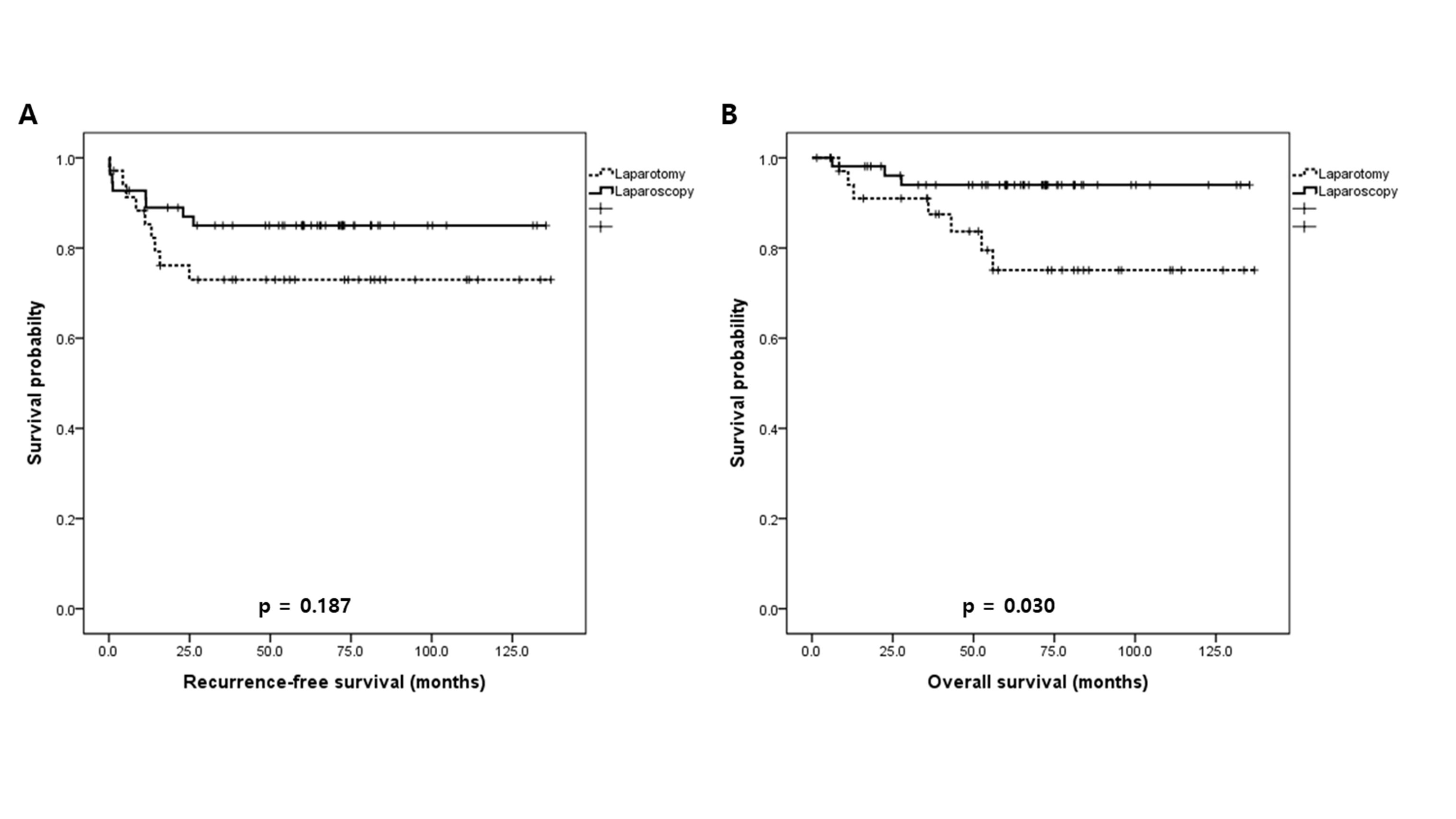

Supplement: Supplementary Material 1 — Kaplan–Meier curves for (A) recurrence-free survival and (B) overall survival by laparoscopy and laparotomy in presumed early-stage, high-risk endometrial cancer patients. [file Image_1.TIF]
